# Association between Average Vitamin D Levels and COVID-19 Mortality in 19 European Countries—A Population-Based Study

**DOI:** 10.3390/nu15224818

**Published:** 2023-11-17

**Authors:** Amar S. Ahmad, Nirmin F. Juber, Heba Al-Naseri, Christian Heumann, Raghib Ali, Tim Oliver

**Affiliations:** 1 Cancer Intelligence, Cancer Research UK, London E20 1JQ, UK; 2 Public Health Research Center, New York University Abu Dhabi, Abu Dhabi P.O. Box 129188, United Arab Emirates; nirmin.juber@nyu.edu; 3 Academic Unit of Medical Education, University of Southampton, Southampton SO17 1BJ, UK; hman1e20@soton.ac.uk; 4 Department of Statistics, Ludwig Maximilian University of Munich, 80539 München, Germany; christian.heumann@stat.uni-muenchen.de; 5 MRC Epidemiology Unit, University of Cambridge, Cambridge CB2 0SL, UK; raghib.ali@mrc-epid.cam.ac.uk; 6 Barts Cancer Institute, Queen Mary University of London, London EC1M 6AU, UK; r.t.oliver@qmul.ac.uk

**Keywords:** population-based study, vitamin D, Europe, COVID-19, coronavirus, mortality, generalized estimation equation model

## Abstract

Early epidemic reports have linked low average 25(OH) vitamin D levels with increased COVID-19 mortality. However, there has been limited updated research on 25(OH) vitamin D and its impact on COVID-19 mortality. This study aimed to update the initial report studying the link between vitamin D deficiency and COVID-19 mortality by using multi-country data in 19 European countries up to the middle of June 2023. COVID-19 data for 19 European countries included in this study were downloaded from Our World in Data from 1 March 2020, to 14 June 2023, and were included in the statistical analysis. The 25(OH) vitamin D average data were collected by conducting a literature review. A generalized estimation equation model was used to model the data. Compared to European countries with 25(OH) vitamin D levels of ≤50 nmol/L, European countries with 25(OH) vitamin D average levels greater than 50 nmol/L had lower COVID-19 mortality rates (RR = 0.794, 95% CI: 0.662–0.953). A statistically significant negative Spearman rank correlation was observed between 25(OH) vitamin D average levels and COVID-19 mortality. We also found significantly lower COVID-19 mortality rates in countries with high average 25(OH) vitamin D levels. Randomized trials on vitamin D supplementation are needed. In the meantime, the issue of vitamin D use should be debated in relation to the ongoing discussions of national post-COVID-19 resilience against future pandemics.

## 1. Introduction

The COVID-19 pandemic is one of major public health concerns due to high mortality rates and hospitalization, particularly among older individuals aged 65 years or above [1,2,3,4]. The impact of this pandemic is known to be disproportionate globally due to younger people’s composition or proportion in populations. In the African region except for South Africa, for example, where the majority of its population is under 65 years of age [5], lower COVID-19 infection and mortality rates were observed compared to most countries worldwide [6,7]. Previous studies have suggested that non-pharmaceutical interventions aimed at controlling COVID-19 transmission, such as through universal lockdowns, were known to be a primary means of reducing COVID-19 case incidence, despite the economic consequences that followed [8,9,10]. By the middle of 2021, COVID-19 had affected nearly 180 million individuals worldwide, whereas the European region reported the highest number of COVID-19 cases [11]. 

Malnutrition is known to be one of the risk factors for increased mortality and morbidity [12]. Nutrition and infectious diseases, for instance, are linked in several ways, including in terms of the development of the human immune system [13]. Previous studies suggested that certain micronutrients, such as vitamin D, played a role in various infectious processes [14]. Vitamin D is a hormone metabolized and coordinated in the skin, liver, and kidney [15]. Vitamin D plays a crucial role in regulating metabolic functions and maintaining skeletal health [16]. Vitamin D is also known to be related to infection and immunity as it can modulate innate and adaptive immune responses [17]. In addition, previous studies have established the connection between vitamin D deficiency and the risk of infectious diseases, including influenza [18,19]. There have been a few observational studies demonstrating a relationship between vitamin D deficiency and COVID-19 infection and mortality [20,21,22,23]. A previous systematic review and meta-analysis study reported better clinical outcomes and mortality rate reduction among COVID-19 patients after receiving replacement therapy with calciferol [24]. However, to date, there have been limited major trials regarding vitamin D and COVID-19 infection. The most recent vitamin D supplementation trials have shown improved liver function among 140 COVID-19 hospitalized patients [25]. 

There has been ongoing research on vitamin D and its impact on COVID-19 infection and mortality in European countries as this region was one of the earliest regions to conduct research on this topic of interest since the early period of COVID-19 back in 2020 [26,27,28]. Furthermore, initial population-based average levels of 25(OH) vitamin D were collected during the first 2 months of the pandemic and found to correlate inversely with COVID-19 mortality [26]. Nevertheless, there is limited updated research on 25(OH) vitamin D deficiency and its impact on COVID-19 mortality. This study aimed to update the initial report studying the link between 25(OH) vitamin D deficiency and COVID-19 mortality rates based on public records of the number of COVID-19 deaths from 1 March 2020, to 14 June 2023. This study included 19 European countries, namely: Portugal, Spain, Switzerland, the United Kingdom (UK), Belgium, Italy, Germany, Austria, Ireland, Greece, The Netherlands, France, Hungary, Czechia, Denmark, Norway, Finland, Sweden, and Slovakia.

## 2. Materials and Methods

### 2.1. Data Sources and Variables

COVID-19 pandemic data for 19 European countries were retrieved from the “Our World in Data” website, which provides real-time statistics on the COVID-19 pandemic, including COVID-19 mortality cases worldwide [29,30]. This source contains data derived from international institutions or statistical agencies, such as from the World Bank and the United Nations [31]. Data from 1 March 2020, to 14 June 2023, were included in the statistical analysis. We selected 19 European countries as a previous study on vitamin D and COVID-19 mortality showed sufficient numbers with regard to population size and the reported number of COVID-19 cases in order to perform suitable analyses with regard to COVID-19 mortality [11]. 

Data on the prevalence of 25(OH) vitamin D deficiency in these 19 European countries were extracted through the literature review that provided vitamin D data by the time we started the analysis [26,32,33]. We used a cut-off of 50 nmol/L for 25(OH) vitamin D, whereas the deficiency range of vitamin D is <50 nmol/L, as it is common in epidemiological studies to make study findings more interpretable and easier to understand by public health professionals [34,35]. The covariates or third variables that we included in our multivariate model were extracted from “Our World in Data” and were selected based on previous studies. These were population age structure [36,37] and year of the COVID-19 pandemic [38]. These variables were known to confound the association between vitamin D and COVID-19 clinical outcomes, including COVID-19 mortality [36,37,38]. 

### 2.2. Statistical Analysis

A Spearman rank correlation was used to evaluate the association between the total number of COVID-19 deaths and the average 25(OH) vitamin D levels of the 19 European countries at five different quantiles (Table 1). A jackknife empirical 95% confidence interval for the Spearman’s correlation was further computed [39]. Whenever vitamin D data were not available, the last observation carried forward was used to impute missing values [40]. In the case of Greece’s 25(OH) vitamin D average data unavailability, the missing values were imputed in terms of the 25(OH) vitamin D median value of all other countries included in the study [26].

To improve the reliability of our study, we used the Poisson GEE model, which enabled us to control for correlation using robust standard errors. This was carried out so that all countries were assigned the same weight and were able to handle different numbers of observations per cluster if one used simple correlation structures. In the first multivariate analysis, a Poison generalized estimation equation (GEE) regression model was fitted with the number of COVID-19 deaths as an outcome. The percentage of those aged 70+ years, the binarized 25(OH) vitamin D, and the year of the COVID-19 pandemic variables were used as fixed-effect predictors. The variable for the 19 European countries was used as a cluster identifier. The population variable was added as an offset in the model. Relative risks (RRs), 95% confidence intervals (95% CI), and corresponding robust z- as well as *p*-values were computed. In the second multivariate analysis, a quasi-Poisson regression model was performed with the total number of COVID-19 deaths as an outcome, and percentage of those aged 70+ years and the average of 25(OH) vitamin D as predictors. The population variable was added as an offset in the model. The number of COVID-19 deaths was predicted for different average 25(OH) vitamin D and population aged 70+ years values. The root mean square error (RMSE) was computed as a measure of goodness of fit as an “in sample” prediction error, since the usual criteria such as AIC or BIC are not available for GEE. In a sensitivity analysis, Greece was excluded and the first multivariate analysis was repeated with data from 18 European countries using a multivariate Poisson GEE regression model. Lastly, four multivariate quasi Poisson regression models were fitted with the Qu.1st (25th quantile), Median, Qu.3rd (75th quantile), and the total COVID-19 deaths (Max) as an outcome. The predictors were percentage of those aged 70+ years and the average of 25(OH) Vitamin D, and the population variable was added as an offset in each model. The linear predictor was plotted for aged 70+ and Vitamin D from the fitted model in the next analysis.. All applied statistical tests were two-sided, and *p*-values < 0.05 were considered statistically significant. Statistical analyses were performed in R 4.2.3 [41].

## 3. Results

Table 1 shows the data used in the statistical analysis in 19 European countries listed in ascending order by 25(OH) vitamin D values. The min. and max. values present the observed COVID-19 death data from March 01, 2020, to June 14, 2023. The first quantile (Qu.1st), median, and third quantile (Qu.3rd) data are summarized as the 25th, 50th (median), and 75th quantile of COVID-19 deaths from 1 March 2020, to 14 June 2023, respectively (Table 1). 

**Figure 1:**
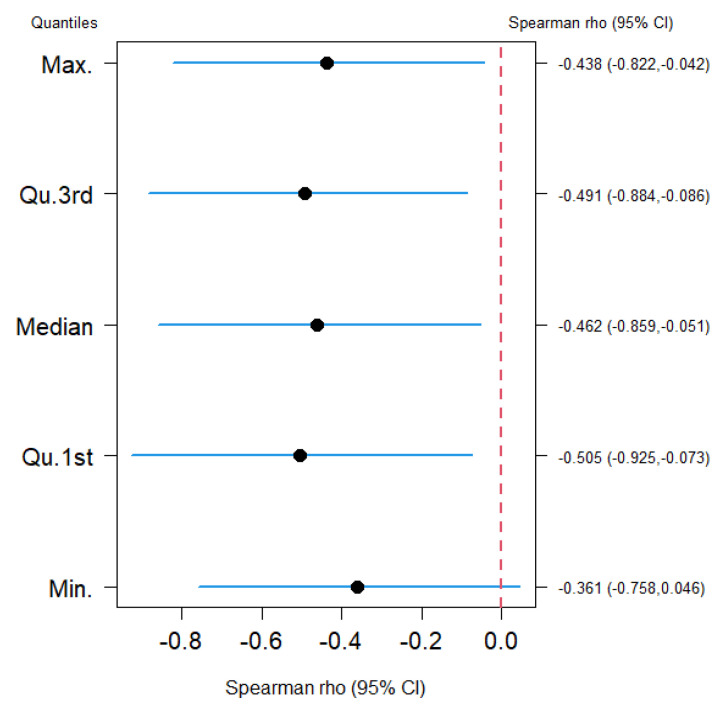
Estimated Spearman’s correlation between the total number of COVID-19 deaths with average 25(OH) vitamin D level at min. (data from 1 March 2020) and max. (data up to 14 June 2023) as well as at the 25th (Qu.1st), 50th (median) and 75th (Qu.3rd) quantiles. A negative and statistically significant Spearman’s correlation was observed (black dots) as all computed 95% confidence intervals (blue lines) do not include zero (red line).

Figure 1 shows the correlation association between the average 25(OH) vitamin D level and the total COVID-19 mortality during the observational period. A statistically significant moderate negative Spearman’s ρ correlation was observed between the average 25(OH) vitamin D level and the total number of COVID-19 deaths at the 25th quantile, median, 75th quantile, and maximum.

Table 2 presents the result from the first fitted multivariate generalized estimation equation Poisson regression model. Countries with a 25(OH) vitamin D average level of >50 nmol/L (20 ng/mL) had lower COVID-19 death rates, as compared with countries with 25(OH) vitamin D average levels of ≤50 nmol/L, after adjusting for population age structure and year of the COVID-19 pandemic with an RR of 0.794 (95% CI: 0.662–0.953, z-value = −2.478, and *p*-value = 0.013). The percentage of those aged 70+ was not statistically significant with an RR of 0.981 (95% CI: 0.926–1.038, z-value = −0.676, and *p*-value = 0.499). There were lower COVID-19 mortality rates in 2021, 2022, and 2023, as compared to 2020 (reference group). However, the difference was only statistically significant in 2022 and 2023, as compared to the reference group in 2020 with an RR of 0.540 (95% CI: 0.428–0.681, z-value = −5.214, and *p*-value < 0.001) and RR of 0.196 (95% CI: 0.154–0.249, z-value = −13.204, and *p*-value < 0.001), respectively. The estimated RMSE for the fitted GEE model in Table 2 was 102.9 compared to an RMSE of 103.9 for a fitted GEE model without 25(OH)-vitamin D level, showing an improvement when the 25(OH) vitamin D level was added to the model.

**Figure 2:**
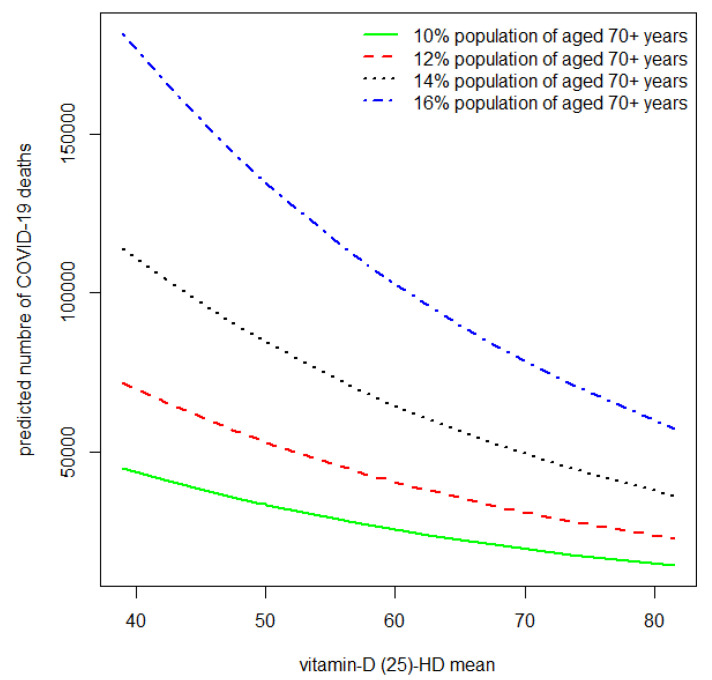
Predicted COVID-19 mortality by 25(OH) vitamin D average for different percentages of age 70+ from a fitted multivariate quasi-Poisson regression model with the total number of COVID-19 deaths on 14 June 2023 as an outcome, and the 25(OH) vitamin D average and age 70+ as predictors. The predicted number of COVID-19 deaths is higher when the 25(OH) vitamin D average level is low, as compared to countries with 25(OH) vitamin D average levels greater than 50. This is applied for different percentages of age 70+. However, the predicted number of COVID-19 deaths was higher for higher percentages of the population aged 70+.

Figure 2 shows the predicted COVID-19 cases from the second fitted multivariate analysis. The predicted number of COVID-19 deaths is higher when the 25(OH) vitamin D average is low as compared to countries with 25(OH) vitamin D averages >50 nmol/L. This is applied to different percentages of those aged 70+ years. However, the predicted number of COVID-19 deaths was higher for higher percentages of those aged 70+ years.

Table 3 presents our sensitivity analysis by excluding Greece in the repeated analysis (data from 18 European countries) using a multivariate Poisson GEE regression model. A similar result in terms of the association between vitamin D deficiency and COVID mortality was shown. 

**Figure 3:**
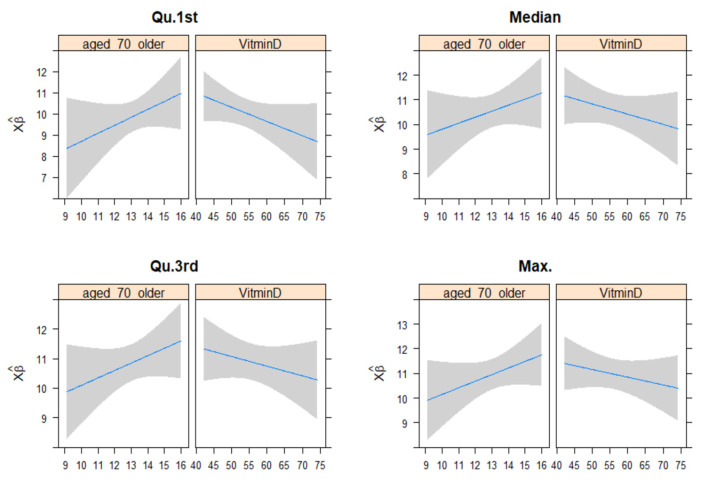
Associations of vitamin D and age 70+ estimated by a multivariate quasi-Poisson regression fitted model with COVID-19 mortality (at the Qu.1st, median, and Qu.3rd, and total death or max which summarized the cumulative COVID-19 mortality over the period from 2 March 2020, to 14 June 2023) as an outcome, and vitamin D as a predictor, stratified by age category of 70+ years. The blue line shows the correlation plot and shaded area is the 95% confidence interval.

Figure 3 presents the associations of vitamin D and age 70+ estimated by a multivariate quasi-Poisson regression fitted model with COVID-19 mortality in four different quantiles. This summarized the cumulative COVID-19 mortality over the period from 2 March 2020, to 14 June 2023) as an outcome, and vitamin D as a predictor, stratified by the age category of 70+ years.

## 4. Discussion

This study provided an updated linkage between 25(OH) vitamin D and COVID-19 mortality rates. In this multi-country study, we found significantly lower COVID-19 mortality rates in countries with the highest average 25(OH) vitamin D levels. In addition, we found statistically significant correlations between vitamin D and COVID-19 mortality at different time points during our observation period. We used the quantile points that were determined based on the distribution of population deaths due to COVID-19 over time, to provide a more granular view of the data and to better understand the distribution of deaths across different periods. In addition, we can account for the size of each country’s population in our analyses, ensuring that our results are not biased towards countries with larger populations. This allows us to make accurate comparisons of COVID-19 impacts across countries of varying population sizes. The 25(OH) vitamin D levels may be linked to COVID-19 outcomes through several mechanisms, including innate and adaptive cellular immunity [17], and renin–angiotensin system regulation [42]; therefore, this suggests that increasing 25(OH) vitamin D concentration may improve the prognosis of COVID-19. Whether vitamin D supplementation is an effective strategy to reduce the risk of COVID-19 clinical outcomes should be further investigated in randomized trials. To date, there are limited published papers on vitamin D and COVID-19 clinical outcomes in randomized trials [43,44,45,46], in spite of the known role of vitamin D in the control of the COVID-19 pandemic. 

We found that European countries with 25(OH) vitamin D average levels greater than >50 nmol/L had lower COVID-19 mortality rates, compared to European countries with 25(OH) vitamin D levels of ≤50 nmol/L. Similar results were found when Greece, the only country with no national data on vitamin D, was excluded in our sensitivity analysis. A systematic review and meta-analysis study by Pereira et al. [45] showed that vitamin D deficiency (<50 nmol/L) was related to higher COVID-19 mortality rates (OR = 1.82, 95% CI = 1.06–2.58). In agreement with the previously mentioned study, a previous hospital-based cohort study involving 185 patients in Germany also showed that vitamin D deficiency was associated with a higher risk of COVID-19 mortality, after adjusting for age, gender, and comorbidities (HR = 14.73, 95% CI = 4.16–52.19 [47]. However, other systematic review and meta-analysis studies by Kummel et al. [43], involving eight RCT studies, and Varikasuvu et al. [46], involving four RCT studies, did not find a statistically significant association between vitamin D supplementation and COVID-19 mortality (OR= 0.74, 95% CI = 0.32–1.71 and RR = 0.78, 95% CI = 0.25–2.40, respectively). The previously stated findings were also consistent with a UK study that utilized Biobank data involving 502,624 participants aged 37–73 years between 2006 and 2010. This study found that those with COVID-19 infection had lower 25(OH) vitamin D levels (median = 43.8, IQR: 28.7–61.6), compared to those without COVID-19 infection (median = 47.2, IQR: 32.7–62.7) [22]. However, the observed statistical significance disappeared after adjustment for confounders.

Our results from a quasi-Poisson regression model by plotting age 70+ years and vitamin D with COVID-19 mortality at each point of the observation period showed positive correlations between age and COVID-19 mortality. We selected the age 70+ population and performed separate analyses using this age categorization, as this primary cutoff has been widely used to reflect the significant demographic shift towards ageing populations as well as to COVID-19 severity [4]. We also observed a negative correlation between vitamin D and COVID-19 mortality in this age-stratified analysis. The possible mechanism of this negative correlation can be described as follows: Vitamin D contributes to improved COVID-19 clinical outcomes and lower mortality as it may enhance the body’s defense against the SARS-CoV-2 virus by stimulating the production of antimicrobial peptides and reducing the inflammatory response [48]. Next, vitamin D deficiency is often associated with several comorbidities, like cardiovascular diseases and diabetes, that are known to increase the severity of COVID-19 [49]. Therefore, adequate levels of vitamin D could indirectly reduce COVID-19 mortality by mitigating these comorbidities. In agreement with the study findings, a previous study found that 25(OH) vitamin D serum deficiency was associated with a risk of death in elderly COVID-19 patients (mean age 76 ± 13 years) [50]. In addition, a previous study on vitamin D supplementation during or just before COVID-19 among the elderly has shown promising results with a lower severity of COVID-19 and a better survival rate [51]. However, our results were different from findings from a UK prospective study that revealed no significant difference in mortality rates between deficient and replete groups [52]. A lower sample size in the specific at-risk age group that led to the study being underpowered to detect differences may explain this finding, compared to the finding in our study, as was stated in the discussion by the authors of this conflicting study [52]. Studies to evaluate vitamin D supplements in elderly COVID-19 patients among the elderly population are therefore worthwhile. On one hand, the elderly are known to be more susceptible to COVID-19 morbidity or mortality due to their poorer health conditions and comorbid conditions [53]. On the other hand, the elderly population has a well-recognized risk of lower 25(OH) vitamin D levels [54], they tend to have reduced vitamin D levels due to higher medication use that may slow vitamin D production [55], as well as having mobility issues that correspond directly to less exposure to sunlight [16]. 

We also found that most northern European countries, despite being less exposed to sunlight, had lower COVID-19 mortality rates compared to the other European countries included in this study. This is an interesting finding due to the fact that sunlight exposure is known to be related to vitamin D production in the body [16]. Previous studies showed the Scandinavian nations have the highest vitamin D levels and lowest COVID-19 mortality, at least in part due to their attention to vitamin D public health education and food fortification policy [56,57]. This raises the ongoing issue of a lack of active monitoring for the detection of vitamin D deficiency in high-risk individuals, such as in the United Kingdom (UK). Despite the fact that the UK Government regularly stated that the consequences of the COVID-19 pandemic would lessen during the summer months as vitamin D levels are high during this season, there has been no funding for clinical trials of vitamin D supplementations, especially after negative reviews that were mainly based on other data by PHE, SACN, and NICE were taken into account [58]. The previously mentioned negative view focuses attention on an ongoing controversy dating back to the 1950s, i.e., whether food should be supplemented with vitamin D. Initially, as shown in the early years after the Second World War, people in the UK showed great enthusiasm for vitamin D supplementation, which led to childhood mortality due to vitamin D toxicity (hypercalcemia). As a result, the UK has been more reluctant to sanction such supplementation, while it has been increasingly used in the Scandinavian countries, such as Norway and Finland [59]. This could explain why most of these countries, in our analysis, despite having less sunshine than the UK, had lower RRs for COVID-19 mortality. The previously stated interpretation was started by Meltzer et al., who studied the link between vitamin D levels and COVID-19 infection among 489 medical center patients in Chicago [60]. Having said this, maintaining a healthy, balanced nutritional status is also important for individuals to overcome potential COVID-19 infection [61]. In addition, the health benefits of safe outdoor activity were also suggested [62], given that at least one of the health benefits of such activity is that it is one of the best ways to raise vitamin D levels [63].

Our study has several limitations. First, this study is prone to residual confounding factors, such as COVID severity and comorbidity [64]. However, a previous study showed that vitamin D deficiency was very prevalent (93.1%) among severe–critical COVID-19 patients, and the 25(OH) vitamin D average was significantly lower among severe–critical COVID-19 patients, compared to moderate COVID-19 patients [65]. The Boston group supported the previously mentioned findings and suggested a link between vitamin D deficiency and increased severity of COVID-19 [66]. In addition, due to vitamin D supplementation in northern European countries [67], the latitude variable was not included in the statistical analysis. Next, the countries’ data used in this study are also not necessarily representative, depending on the 25(OH) vitamin D data collection in each country. Due to the observational nature of our study, we could not provide mechanistic information with regard to the effect of vitamin D supplementation and COVID-19 mortality. Consequently, our study findings could not imply causation, despite an observed correlation between low vitamin D levels and high COVID-19 mortality. To the best of our knowledge, there is no data from the UK on the effect of vitamin D supplementation, despite an ongoing debate since March 2020 regarding vitamin D deficiency that disproportionally affects the black, Asian and minority ethnic (BAME) population as reflected by excess COVID-19 mortality in this specific community [68]. Next, no COVID-19 data by gender or race/ethnicity were available at the time of this analysis, which is considered a limitation in the statistical analysis. However, it should be noted that the majority of these 19 European countries are white Caucasians. Lastly, our study is subject to residual confounding factors, such as COVID-19 severity [35,69] and comorbidity [22]. 

## 5. Conclusions

Our study showed a strong and statistically significant association between vitamin D deficiency (≤50 nmol/L) and the total number of COVID-19 deaths in 19 European countries from 1 March 2020, to 14 June 2023. We further found significantly lower COVID-19 mortality rates in countries with high average 25(OH) vitamin D levels. However, this population study cannot suggest the role of vitamin D during COVID-19 to allow us to make any clinical decisions. Longitudinal studies and randomized control trials to better reveal the role of vitamin D and COVID-19 clinical outcomes are warranted. In the meantime, the issue of vitamin D use should be debated in relation to the ongoing discussions of national post-COVID-19 resilience against future pandemics.

## Figures and Tables

**Table 1:** Data used in the statistical analysis in 19 European countries. Min. represents COVID-19 mortality on 1 March 2020 and max. represents COVID-19 mortality on 14 June 2023. The Qu.1st, median, and Qu3rd, determined based on the distribution of population deaths due to COVID-19 over time, are summarized in terms of the cumulative COVID-19 mortality over the period from 2 March 2020, to 14 June 2023. The population of each country included all ages. Age 70+ represents the proportion of the population aged 70 years and older in 2015, as stated in Our World in Data, and 25(OH) vitamin D is presented as country-level data. Data are listed in ascending order by vit. D values.

Country/ Location	Min.	Qu.1st	Median	Qu.3rd	Max.	Population	Age 70+ Population	25(OH) Vit D (nmol/L)	25(OH) Vit D (ng/mL)
Portugal	1	6490	18,109	24,805	26824	10,270,857	14.924	39.00	15.63
Spain	1	53,606	89,004	114,946	121,416	47,558,632	13.799	42.50	17.03
Switzerland	2	7085	10,800	13,540	14,020	8,740,471	12.644	46.00	18.43
United Kingdom	3	89,820	166,690	206,036	226,977	67,508,936	12.527	47.40	18.99
Belgium	1	19,341	25,984	32,449	34,360	11,655,923	12.849	49.30	19.75
Italy	29	71,359	131,724	174,300	190,625	59,037,472	16.24	50.00	20.03
Germany	1	45,885	97,611	150,593	174,545	83,369,840	15.957	50.10	20.07
Austria	1	6979	13,658	20,513	22,518	8,939,617	13.748	56.00	22.44
Ireland	1	2228	5492	7927	8998	5,023,108	8.678	56.40	22.60
Greece	1	4507	15,519	32,077	37,052	10,384,972	14.524	57.95	23.22
The Netherlands	1	10,891	18,277	22,564	22,992	17,564,020	11.881	59.50	23.84
France	2	62,051	115,014	149,846	163,787	67,813,000	13.079	60.00	24.04
Hungary	1	8951	30,492	47,083	48,790	9,967,304	11.976	60.60	24.28
Czechia	1	11,242	30,707	40,807	42,806	10,493,990	11.580	62.50	25.04
Denmark	1	1140	2696	6833	8736	5,882,259	12.325	65.00	26.04
Norway	1	421	929	3958	5556	5,434,324	10.813	65.00	26.04
Finland	1	574	1199	5782	9798	5,540,745	13.264	67.70	27.12
Sweden	1	9229	14,999	19,796	24,391	10,549,349	13.433	73.50	29.45
Slovakia	1	1732	12,886	20,322	21,167	5,643,455	9.167	81.50	32.65

**Table 2:** Multivariate generalized estimation equation Poisson regression model with the percentage of age 70+, binarized vitamin D, and year variables as fixed-effect predictors, and COVID-19 deaths as outcome. The country variable was used as cluster identifier. The population variable was added as an offset.

	Multivariate	
Variable	RR (95% CI)	Robust z-Value (*p*-Value)
25(OH) vitamin D ≤ 50 nmol/L	1.000 (Reference Group)	
25(OH) vitamin D > 50 nmol/L	0.794 (0.662, 0.953)	−2.478 (0.013)
Age 70+ years in population	0.981 (0.926, 1.038)	−0.676 (0.499)
Year 2020	1.000 (Reference Group)	
Year 2021	0.878 (0.720, 1.072)	−1.274 (0.203)
Year 2022	0.540 (0.428, 0.681)	−5.214 (<0.001)
Year 2023	0.196 (0.154, 0.249)	−13.204 (<0.001)

**Table 3:** Multivariate generalized estimation equation Poisson regression model from 18 countries (excluding Greece) with the percentage of age 70+, binarized vitamin D, and year variables as fixed-effect predictors, and COVID-19 deaths as outcome. The country variable was used as a cluster identifier. The population variable was added as an offset.

	Multivariate	
Variable	RR (95% CI)	Robust z-Value (*p*-Value)
25(OH) vitamin D ≤ 50 nmol/L	1.000 (Reference Group)	
25(OH) vitamin D > 50 nmol/L	0.780 (0.653, 0.931)	−2.750 (0.006)
age 70+	0.977 (0.924, 1.032)	−0.840 (0.401)
Year 2020	1.000 (Reference Group)	
Year 2021	0.878 (0.715, 1.078)	−1.241 (0.215)
Year 2022	0.525 (0.418, 0.659)	−5.545 (<0.001)
Year 2023	0.204 (0.154, 0.269)	−11.188 (<0.001)

## Data Availability

The datasets generated during and/or analyzed in this study are available on the Our World in Data repository, which can be found at: https://ourworldindata.org/coronavirus#explore-the-global-situation (accessed on 15 June 2023).
